# Clinical Relevance of Topical Antibiotic Use in Coselecting for Multidrug-Resistant Staphylococcus aureus: Insights from *In Vitro* and *Ex Vivo* Models

**DOI:** 10.1128/AAC.02048-20

**Published:** 2021-04-19

**Authors:** Yi Nong, George Taiaroa, Shivani Pasricha, Romain Guérillot, Ian R. Monk, Sarah L. Baines, Glen P. Carter, Benjamin P. Howden, Deborah A. Williamson

**Affiliations:** a Department of Microbiology and Immunology at the Peter Doherty Institute for Infection and Immunity, The University of Melbourne, Melbourne, Australia; b Microbiological Diagnostic Unit Public Health Laboratory, Department of Microbiology & Immunology, The University of Melbourne at The Doherty Institute for Infection and Immunity, Melbourne, Australia; c Doherty Applied Microbial Genomics, Doherty Institute, The University of Melbourne, Melbourne, Australia; d Department of Microbiology, Royal Melbourne Hospital, Melbourne, Australia

**Keywords:** *Staphylococcus aureus*, coselection, fusidic acid, multidrug resistance, mupirocin, topical antibiotic

## Abstract

Topical antibiotic preparations, such as fusidic acid (FA) or mupirocin, are used in the prevention and treatment of superficial skin infections caused by staphylococci. Previous genomic epidemiology work has suggested an association between the widespread use of topical antibiotics and the emergence of methicillin-resistant Staphylococcus aureus in some settings. In this study, we provide experimental proof of coselection for multidrug resistance in S. aureus following exposure to FA or mupirocin. Through targeted mutagenesis and phenotypic analyses, we confirmed that *fusC* carriage confers resistance to FA and *mupA* carriage confers high-level resistance to mupirocin in multiple S. aureus genetic backgrounds. *In vitro* experiments demonstrated that carriage of *fusC* and *mupA* confers a competitive advantage in the presence of subinhibitory concentrations of FA and mupirocin, respectively. Further, we used a porcine skin colonization model to show that clinically relevant concentrations of topical antibiotics can coselect for the presence of unrelated antimicrobial resistance determinants, such as *mecA*, *blaZ*, and *qacA*, in *fusC-* or *mupA*-harboring S. aureus. These findings provide valuable insights on the role of acquired FA or mupirocin resistance in coselecting for broader antibiotic resistance in S. aureus, prompting a greater need for judicious use of topical antibiotics.

## Abstract (precis)

Topical antibiotic preparations, such as fusidic acid (FA) or mupirocin, are used in the prevention and treatment of superficial skin infections caused by staphylococci. Previous genomic epidemiology work has suggested an association between the widespread use of topical antibiotics and the emergence of methicillin-resistant Staphylococcus aureus in some settings.

## INTRODUCTION

Skin and soft-tissue infections (SSTIs) caused by Staphylococcus aureus are among the most common bacterial infections worldwide (1). Topical antibiotics, such as fusidic acid (FA) and mupirocin, are widely used in some settings for both the prevention and treatment of such infections (2). The former has been used as a first-line topical treatment option for superficial SSTIs (e.g., impetigo) in many countries outside the United States (3, 4). Mupirocin, as a monotherapy or in combination with skin antiseptics such as chlorhexidine, is used in the prevention of surgical site infections and in preoperative clearance of methicillin-resistant S. aureus (MRSA) (5, 6). However, following recent increases in resistance to these antibiotics, there is concern for the potential for “collateral damage” associated with the use and misuse of these agents and coselection of multidrug-resistant (MDR) S. aureus. For example, high population-level use of FA and mupirocin in New Zealand not only led to elevated levels of S. aureus resistance to these two agents but also selected for the emergence of MRSA lineages (7). Comparative genomic analyses of these isolates showed that FA resistance was mediated by the *fusC* gene, carried on mobile staphylococcal cassette chromosome (SCC) elements either with or without the methicillin resistance determinant *mecA* (8, 9). The colocalization of *fusC* and *mecA* on SCC elements suggested the genetic potential for coselection of MRSA driven by FA exposure. Further, resistance to mupirocin was mediated by the *mupA* gene, located on a nonconjugative plasmid (pNZAK1), which also harbored genes associated with increased tolerance to chlorhexidine (*qacA*) and penicillin resistance (*blaZ*), highlighting the potential for coselection of other resistance mechanisms (9).

In other parts of the world, the widespread use of topical antibiotics has also been linked to an increased prevalence of acquired resistance to both topical and systemic antimicrobials in S. aureus. As examples, *fusC* was the most prominent FA resistance mechanism in Australia (10), Taiwan (11), and several European countries (12–14). This coincided with the emergence of community-associated MRSA harboring novel transferable SCC*mec-fusC* gene cassettes (11, 13, 14). Similarly, increasing rates of high-level mupirocin-resistant S. aureus, often conferred by plasmid-borne *mupA*, have been reported in settings where mupirocin use was common (15–17). Of particular concern are reports of nasal colonization of patients with *mupA*-harboring MRSA in intensive care units, highlighting the potential for subsequent failure of decolonization (17). As with *fusC*, the cooccurrence of *mupA* with resistance determinants to macrolides, gentamicin, and tetracycline on the same plasmids may have important clinical implications (18). Accordingly, the aim of this study was to determine the role of (i) *fusC*-mediated FA resistance and (ii) *mupA*-mediated mupirocin resistance in the coselection of other kinds of drug resistance in S. aureus, with a focus on methicillin resistance. In addition, we developed an *ex vivo* porcine skin colonization model to evaluate the potential selection pressure that topical agents exert on S. aureus at clinically used concentrations on the skin. Collectively, these data provide valuable insights into understanding the potential clinical impact of topical antibiotic resistance on coselection for drug-resistant S. aureus.

## RESULTS

### *fusC* or *mupA* deletions result in loss of resistance to topical antibiotics.

Unmarked deletions of (i) *fusC* in strains NZ14487 (sequence type 1 methicillin-susceptible S. aureus [ST1 MSSA]), NZ14132 (ST1 MRSA), and NZAK3 (ST5 MRSA) or (ii) *mupA* in NZ14487 and NZ14132 were performed by targeted mutagenesis using plasmid pIMAY-Z (19). Subsequently, a similar approach was used to complement the isogenic *fusC* and *mupA* mutants (see Materials and Methods). Each genetic modification made, including unmarked deletions and complementations, was confirmed by PCR and whole-genome sequencing (WGS), with these data showing the S. aureus isolates were otherwise isogenic to the original wild-type strains, lacking secondary mutations that can be acquired during the process of targeted mutagenesis (see Table S1 in the supplemental material).

To confirm the role of *fusC* and *mupA* in mediating FA and mupirocin resistance, respectively, broth microdilution (BMD) MIC assays were performed in accordance with the Clinical and Laboratory Standards Institute (CLSI) guidelines (20). Results were interpreted according to the CLSI breakpoints for mupirocin (20) and the European Committee on Antimicrobial Susceptibility Testing (EUCAST) breakpoints for FA (21), as there are no CLSI-defined breakpoints for FA. Comparison between wild-type and corresponding isogenic *fusC* mutants revealed a 6-log_2_ reduction in FA MICs (from 4 to 0.0625 mg/liter) (Table S2). Similarly, deletion of *mupA* led to a 12-log_2_ reduction in mupirocin MIC (from >1,024 to 0.25 mg/liter) (Table S2). Complementation of *fusC* and *mupA* in these mutants returned the observed FA and mupirocin MICs to wild-type levels (4 and >1,024 mg/liter, respectively), confirming that the changes in phenotype were a direct result of the gene deletions.

### Exposure to sub-MIC levels of FA or mupirocin coselects for MDR S. aureus.

**FIG 1:**
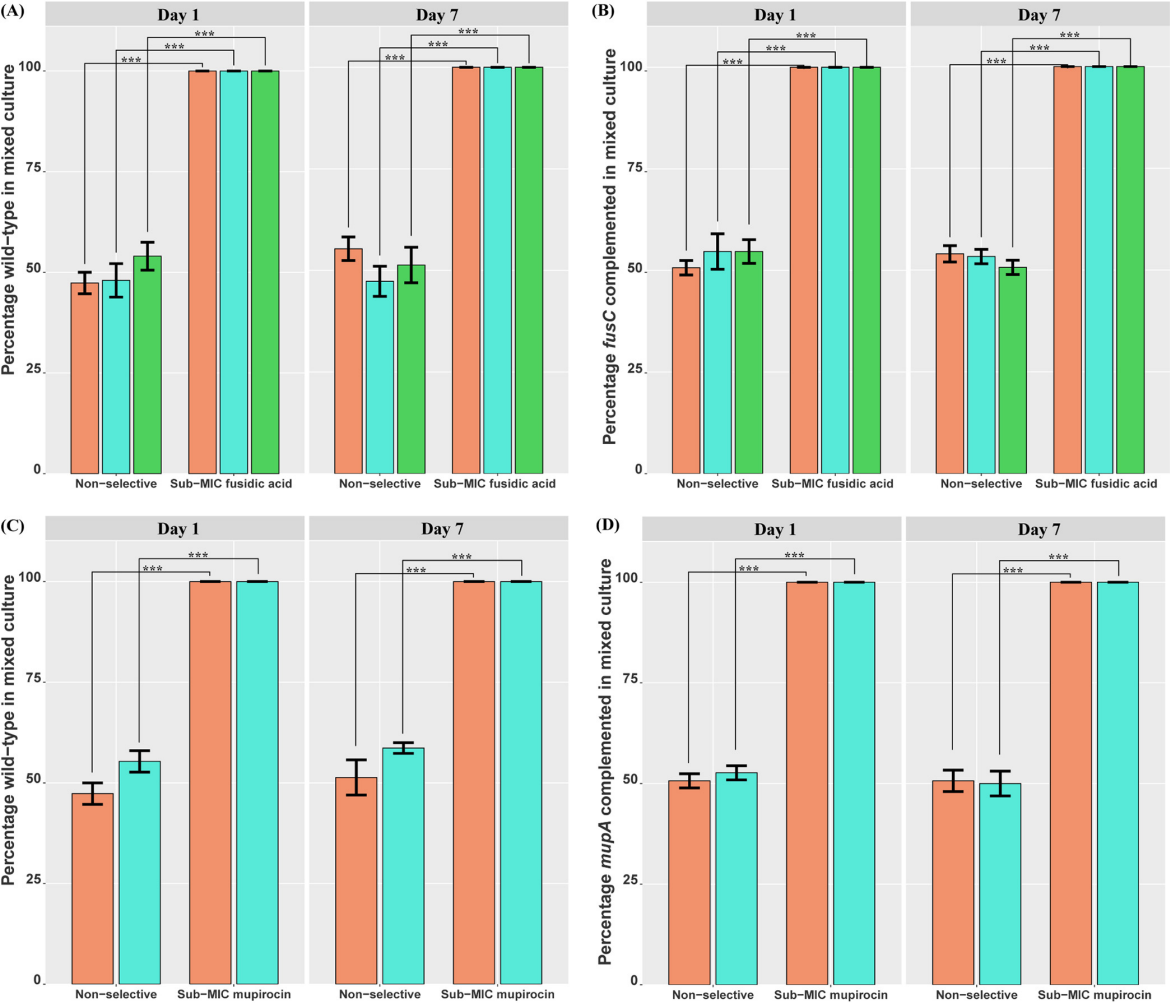
*In vitro* competition assays of S. aureus reveal the selective advantage of topical antibiotic resistance gene carriage. S. aureus strains NZ14132 (orange), NZ14487 (aqua), and NZAK3 (green), wild-type or complemented strains, were paired with their respective isogenic mutants under nonselective conditions and exposure to a sub-MIC level of FA (0.03125 mg/liter) or mupirocin (0.125 mg/liter) for 7 days. (A to D) Percentages of wild-type or complemented isolates in mixed cultures of the wild type and *fusC* mutant (A), *fusC* complemented and *fusC* mutant (B), wild-type and *mupA* mutant (C), and *mupA* complemented and *mupA* mutant (D) strains were determined on days 1 and 7 postexposure. The mean percentages of three biological replicates are displayed for each condition tested, with black error bars representing the standard errors of the means (SEM). Statistically significant differences are indicated by asterisks (***, *P < *0.001 by paired *t* test).

The representative S. aureus isolates used in this study were defined as MDR, i.e., resistance to FA, penicillin, and mupirocin in NZ14487; resistance to FA, penicillin, mupirocin, and oxacillin in NZ14132; and resistance to penicillin, oxacillin, and FA in NZAK3. To determine whether selective pressure exerted by FA or mupirocin exposure coselected for MDR S. aureus, competition assays were performed using wild-type or complemented strains mixed with isogenic mutants in a 1:1 ratio. These assays were conducted in the presence and absence of sub-MIC levels of FA (0.03125 mg/liter) or mupirocin (0.125 mg/liter) *in vitro*, which ensured the viability of mutant strains during antibiotic exposure. Exposure to FA or mupirocin in competition assays rapidly enriched for the wild-type or complemented strains over deletion mutants, with 100% of the isolates being wild-type or complemented strains on day 1 and day 7 postexposure (Fig. 1). No significant difference in the ratio of wild-type or complemented strains compared to mutant strains was observed on day 1 or 7 under nonselective conditions (Fig. 1). Further, growth assays showed no significant difference in doubling time was observed when comparing the complemented and isogenic mutant strains to their respective wild-type strains (Fig. S1), indicating that the selection in the presence of antibiotics was not due to a difference in growth rate. These data suggest that *fusC* and *mupA* play a role in selecting for MDR S. aureus following exposure to sub-MIC levels of FA and mupirocin, respectively.

### Clinically relevant concentrations of topical antibiotics coselect for MDR S. aureus in an *ex vivo* model of skin colonization.

Porcine skin has been used as an experimental model of human skin, given similarities in anatomy, physiology, and morphology (22). Here, we hypothesized that topical application of FA as Fucidin ointment (2%, wt/wt, sodium fusidate) would select for *fusC-*harboring MDR S. aureus wild-type or complemented strains of NZ14487, NZ14132, and NZAK3 grown in competition with isogenic *fusC* mutants on porcine skin. Similarly, we hypothesized that the topical application of mupirocin as Bactroban ointment (2%, wt/wt, mupirocin) would select for *mupA*-carrying wild-type or complemented strains of NZ14487 and NZ14132 grown in competition with isogenic *mupA* mutants.

**FIG 2:**
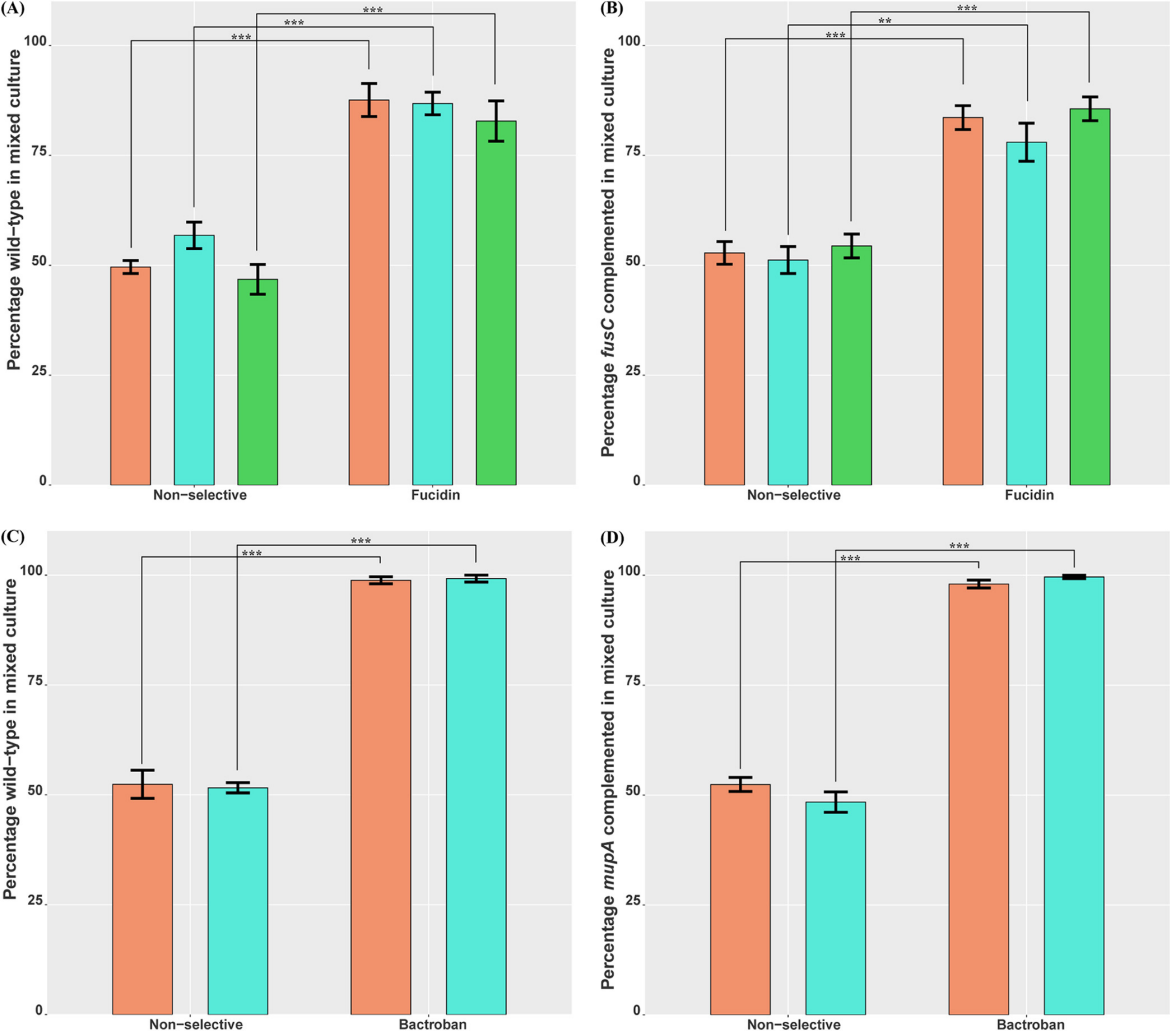
*Ex vivo* competition assays of S. aureus reveal the selective advantage of topical antibiotic resistance gene carriage in clinically relevant environments. S. aureus strains NZ14132 (orange), NZ14487 (aqua), and NZAK3 (green) wild-type or complemented strains paired with their respective isogenic mutants were grown on porcine skin under nonselective conditions and exposure to a single dose of 20 to 25 mg 2% Fucidin or 2% Bactroban ointment for 24 h. (A to D) Percentages of wild-type or complemented isolates within mixed cultures of wild-type and *fusC* mutant (A), *fusC* complemented and *fusC* mutant (B), wild-type and *mupA* mutant (C), and *mupA* complemented and *mupA* mutant (D) strains were determined at the conclusion of the assays. Five biological replicates were used to calculate the mean percentages and the SEM (black error bars) for each condition tested. Statistically significant differences are indicated by asterisks (**, *P < *0.01; ***, *P < *0.001, paired *t* test).

Similar to our *in vitro* results, exposure to Fucidin ointment selected for wild-type and complemented strains over isogenic *fusC* mutants. Specifically, after 24 h of Fucidin treatment, NZ14487, NZ14132, and NZAK3 wild-type strains accounted for 86.8%, 87.6%, and 82.8%, respectively, and their *fusC* complemented strains accounted for 78%, 83.6%, and 85.6% of isolates collected. These percentages were significantly higher than their percentages under nonselective conditions (Fig. 2A and B). In addition, significant enrichment of *mupA*-carrying isolates was observed for the mupirocin *ex vivo* experiments, with 99.2% and 98.8% of the isolates harvested being wild-type NZ14487 and NZ14132, respectively, and 99.6% and 98.0% of isolates being the NZ14487 and NZ14132 *mupA* complemented strains, respectively, following 24 h of exposure to Bactroban (Fig. 2C and D). Conversely, these levels of enrichment for *fusC-* or *mupA*-harboring isolates were not observed under nonselective conditions. For each pairing, five postexposure resistant isolates were randomly selected for WGS, which confirmed the identity of input wild-type or complemented strains and also showed that the horizontal mobilization of *fusC* or *mupA* had not occurred (Table S1).

## DISCUSSION

In this study, we provide experimental evidence of coselection following the topical use of fusidic acid or mupirocin in S. aureus. This included targeted mutagenesis and phenotypic analyses in multiple S. aureus genetic backgrounds, confirming that *fusC* carriage confers resistance to fusidic acid and that *mupA* carriage confers resistance to mupirocin. Further, we used *in vitro* and skin colonization models to determine that clinically relevant concentrations of topical antibiotics are sufficient to enrich for *fusC* or *mupA* harboring S. aureus. These findings are in line with previous genomic studies that highlighted the potential role of coselection following topical antibiotic exposure in the emergence of MDR S. aureus (8, 9). Our findings have several clinical implications when considering the widespread use of topical agents in the prevention and treatment of SSTIs caused by S. aureus.

First, although the colocalization of resistance determinants to topical antibiotics and other antimicrobials has been frequently described in the literature (23–25), experimental assessments of the role of colocalization in facilitating coselection are far less common. With the widespread use of topical antibiotics, an understanding of the coselective potential of their use becomes critical for antibiotic stewardship and for controlling the further spread of MDR S. aureus, including MRSA. Our *in vitro* and on-skin competition assays demonstrate that rapid enrichment of MDR S. aureus can be driven by a single exposure to topical antibiotics if resistance determinants are colocated with *fusC* or *mupA*. Although the growth of susceptible S. aureus mutant strains was partially impaired following exposure to sub-MIC levels of topical antibiotics, this exposure placed a strong selection on the carriage of topical antibiotic resistance genes. Clinically, residual topical antibiotics can be found at very low levels on skin following treatment (26), potentially providing a sub-MIC niche for the selection of antimicrobial resistance.

Second, to date, there are no experimental data assessing whether clinically used concentrations of topical antibiotics can select for MDR S. aureus. Importantly, the concentration of active ingredients in topical preparations of fusidic acid and mupirocin are several orders of magnitude higher than inhibitory MIC levels, with 2% (wt/wt) equivalent to approximately 20,000 mg/liter. Previous colonization models, both *ex vivo* and *in vivo*, have shown that the complete or near-complete eradication of S. aureus can be achieved when these topical ointments are applied shortly after bacterial inoculation on skin (27–29). As such, to appropriately evaluate the selective pressures imposed by clinically relevant concentrations of topical antibiotic, we developed an *ex vivo* porcine skin model of colonization. Although we observed a 4- to 5-log reduction in the number of CFU (unpublished observation), wild-type, complemented, and mutant strains were recovered following exposure to clinically relevant concentrations of topical antibiotics in our model. This suggests that the antimicrobial activity of topical antibiotic preparations is dependent on several factors, such as bacterial load, growth phase, and biofilm formation on skin. Of particular concern is the apparent ability of topical antibiotics to coselect for MRSA isolates in the absence of exposure to a β-lactam class antibiotic and highlight the importance of exploring alternative topical agents, such as the use of hydrogen peroxide, for which acquired tolerance has not been reported (2).

A limitation of this study is that we did not examine the effect of repeated topical antibiotic exposures; it is possible that regular doses of topical antibiotic further reduce bacterial load or decolonize S. aureus on skin (30, 31). Moreover, the translation of our *in vitro* and *ex vivo* findings to inform clinical practice may be better informed in the future by assessments of the effect of host immunity on clearance and the influence of undefined skin microbiota. As such, future work should address these clinical questions using relevant *in vivo* models and clinical studies of S. aureus colonization and infection.

Taken together, our findings provide insights on the molecular basis of topical antibiotic resistance and the potential for this to enable coselection of broader antibiotic resistance in S. aureus. This highlights the need for the judicious use of topical antibiotics and improved surveillance of topical antibiotic resistance to control the spread of antimicrobial resistance.

## MATERIALS AND METHODS

### Bacterial strains and antimicrobial agents.

S. aureus strains NZ14487, NZ14132, and NZAK3 were obtained from previously published studies (8, 9). Unless otherwise stated, all S. aureus isolates were maintained on brain heart infusion (BHI) agar and grown in BHI broth at 37°C with shaking at 200 rpm. FA and mupirocin used for *in vitro* assays were purchased from Sigma-Aldrich, Australia. For *ex vivo* experiments, Fucidin ointment containing 2% (wt/wt) sodium fusidate was purchased from LEO Pharma Pty. Ltd., Australia. APO-mupirocin (Bactroban) ointment containing 2% (wt/wt) mupirocin was obtained from Apotex Pty. Ltd., Australia. Antimicrobial susceptibility testing was performed by broth microdilution assays in accordance with the CLSI guideline (20), and results were interpreted based on the CLSI breakpoints for mupirocin (20) and EUCAST breakpoints for FA (21).

### Construction of isogenic *fusC* or *mupA* mutants by allelic exchange.

The pIMAY-Z shuttle vector (19) was used to make unmarked chromosomal deletions of *fusC* in NZ14487, NZ14132, and NZAK3 strains or *mupA* in NZ14487 and NZ14132 strains. Using the primers listed in Table S3 in the supplemental material, spliced overlap extension (SOE) PCR was used to generate a deletion cassette containing jointed flanking regions (700 to 750 bp) upstream and downstream of the target gene. The amplified cassette was then cloned into pIMAY-Z by seamless ligation cloning extract (SLiCE) (32). To bypass the S. aureus restriction barrier, a deletion plasmid was electroporated into Escherichia coli IM01B or IM08B to obtain methylation profiles similar to that of ST1 or ST5 S. aureus, respectively. The presence of the desired deletion plasmid was confirmed by colony PCR using the flanking primers. Purified plasmid was then introduced into S. aureus by electroporation. Successful transformants were selected on BHI agar supplemented with 10 mg/liter chloramphenicol (CHL) and 100 mg/liter X-Gal (5-bromo-4-chloro-3-indolyl-d-galactopyranoside; Melford) and grown at 30°C for 2 days. Allelic exchange mutagenesis was performed as previously described by Monk et al. (19). To confirm that plasmid loss resulted from double-crossover recombination, white colonies were cross-patched onto selective BHI agar containing Cm and X-Gal as well as nonselective BHI agar. Colony PCR was performed using primers FUSC-OUT-Fp and FUSC-OUT-Rp (*fusC*) or MUPA-OUT-Fp and MUPA-OUT-Rp (*mupA*) to screen CHL-sensitive colonies for chromosomal integration of deletion cassettes. Isogenic mutants were generated for each target gene in the representative strains. Finally, both wild-type and isogenic mutant isolates were subjected to WGS performed on the Illumina NextSeq platform using 2 × 150-bp paired-end chemistry. Deletions of the target genes were visualized by mapping the Illumina reads of mutant strains to their respective wild-type genomes using Geneious v.11.1.5. Snippy v.4.6.0 (https://github.com/tseemann/snippy) was used to detect secondary mutations introduced during the allelic exchange experiments.

### Complementation of *fusC* or *mupA* mutants.

Complementary primers (Table S3) were used to generate complementation cassettes by SOE PCR. The complemented *fusC* strain carried a substitution at nucleotide 237 (c.237G>C), resulting in a silent mutation at codon 79 (p.Val79Val). Similarly, the complemented *mupA* harbored a substitution at nucleotide 1509 (c.1509T>G), causing a silent mutation at codon 503 (p.Ser503Ser). Subsequently, the *fusC* or *mupA* mutant isolates were transformed with pIMAY-Z containing a corresponding complementation cassette as described above. The resulting complemented isolates were subjected to whole-genome sequencing to confirm the integration of complementation cassettes into the chromosome or relevant plasmid, and the presence of secondary mutation-introduced complementation was explored using Snippy (v.4.6.0) (https://github.com/tseemann/snippy).

### Determination of bacterial growth rates.

Growth assays and analyses were performed as previously described in Guérillot et al. (33). A total of six biological replicates were performed for each strain tested. For each replicate, an overnight BHI broth culture of S. aureus was diluted in fresh BHI broth to obtain a bacterial suspension with a starting optical density at 600 nm (OD_600_) of 0.05, and then 200 μl of the bacterial suspension was dispensed into the wells of a 96-well tray. The bacterial cultures were incubated at 37°C for 16 h with agitation, and the OD_600_ was measured every 15 min using a CLARIOstar microplate reader (BMG LABTECH). The bacterial growth rates denoted as doubling times were determined using the R package *cellGrowth* (34). A series of unpaired *t* tests were used to determine statistical significance.

### *In vitro* pairwise competition assays.

For each pairing of the wild-type and mutant strains or the complemented and mutant strains, an overnight broth culture of each strain was diluted in fresh BHI broth to obtain a bacterial suspension at an adjusted OD_600_ of 0.10. The two normalized bacterial cultures were then mixed at a 1:1 ratio. The coculture of competitor strains was diluted 1:100 in 10 ml nonselective BHI broth or BHI broth containing 0.5× MIC of FA or mupirocin (i.e., 0.03125 mg/liter for FA or 0.125 mg/liter for mupirocin) for mutants. The cultures were then incubated at 37°C with shaking at 200 rpm for 7 days. Following 24 h of exposure to antibiotics, 10-fold serial dilutions of a 300-μl sample removed from each broth culture was performed in phosphate-buffered saline (PBS). Samples of 100 μl of appropriate dilutions were spread onto BHI agar plates, which were then incubated at 37°C overnight. The following day, 50 randomly selected single colonies were cross-patched onto antibiotic (FA or mupirocin at 2 mg/liter) containing BHI agar and nonselective BHI agar. The agar plates were then incubated at 37°C overnight before the ratio of the two competing bacterial strains on day 1 was quantified. The process was repeated on day 7 postexposure to determine changes in the bacterial population over time under selective and nonselective conditions. A series of paired *t* tests were used to determine statistical significance.

### *Ex vivo* pairwise competition assay.

Sections of fresh porcine skin were disinfected with 80% ethanol for 30 min, followed by three rinses with PBS. Sections were dried and then coinfected using wild-type or complemented strains paired with isogenic mutants of S. aureus (in equal numbers) at 10^6^ CFU/ml and incubated for 24 h at 37°C to allow bacterial growth on the skin. Following this, topical ointment (20 to 25 mg) or vehicle (deionized water) alone was applied and mixed with bacteria grown on the infected sections (2 by 2 cm^2^), which were then incubated for an additional 24 h at 37°C. Following the incubation, bacterial growth on the skin was collected by suspending the skin sections in PBS for both untreated and treated replicates. The bacterial suspension was then diluted and plated onto nonselective BHI agar plates before the ratio of wild-type or complemented strains to isogenic mutants was determined under selective and nonselective conditions as described above. Five representative isolates were further analyzed by WGS to confirm bacterial identification for each pairing.

### Data availability.

Sequence data for all isolates used in this study have been deposited under the BioProject accession number PRJNA412108 at the National Centre for Biotechnology Information database. Genome assemblies for NZ14487 and NZ14132 have also been deposited under the same accession number, and the NZAK3 complete genome can be accessed using GenBank accession number GCA_900017775.1.

## Supplementary Material

Supplemental file 0

Supplemental file 0
