# Supplementary material for: Clinical Relevance of Topical Antibiotic Use in Coselecting for Multidrug-Resistant Staphylococcus aureus: Insights from In Vitro and Ex Vivo Models
Source: Antimicrob Agents Chemother. 2021 Apr 19;65(5):e02048-20. doi: 10.1128/AAC.02048-20 (PMC8092865; doi:10.1128/AAC.02048-20)
Supplement: Supplemental file 0 [file AAC.02048-20-s000S2.pdf]

SUPPLEMENTARY DATA

Supplementary Table 2 – Phenotypic antibiotic susceptibilities of *S. aureus* wild-type, mutant and complemented strains to fusidic acid (FA) and mupirocin as determined by broth micro-dilution assays in this study.

Strain	WGS ID	FA MIC (mg/L) / Log_2 fold-change relative to wild-type MIC	Mupirocin MIC (mg/L) / Log_2 fold-change relative to wild-type MIC
NZ14132	DW-38-S034-SA	4 / 0	>1024 / 0
NZ14132*fusC*	DW-38-S035-SA	0.0625 / -6	ND*
NZ14132*fusC*::*fusC*	DW-38-S001-SA	4 / 0	ND
NZ14132*mupA*	DW-38-S036-SA	ND	0.25 / -12
NZ14132*mupA*::*mupA*	DW-38-S005-SA	ND	>1024 / 0
NZ14487	DW-38-S047-SA	4 / 0	>1024 / 0
NZ14487*fusC*	DW-38-S048-SA	0.0625 / -6	ND
NZ14487*fusC*::*fusC*	DW-38-S060-SA	4 / 0	ND
NZ14487*mupA*	DW-38-S049-SA	ND	0.25 / -12
NZ14487*mupA*::*mupA*	DW-38-S007-SA	ND	>1024 / 0
NZAK3	DW-38-S061-SA	4 / 0	ND
NZAK3*fusC*	DW-38-S062-SA	0.0625 / -6	ND
NZAK3*fusC*::*fusC*	DW-38-S003-SA	4 / 0	ND

*Abbreviations: ND, not determined.

Supplementary Table 3 – Primer sequences used in the study

Primer	Sequence (5' to 3' direction)	Description
FUSC-Fp	CCTCACTAAAGGGAACAAAAGC TGGGTACCAATAAAATAATGGTG CTTGGGAAAGAAAAG	Flanking primers used for amplification of gene cassettes for *fusC* deletion and complementation
FUSC-Rp	CGACTCACTATAGGGCGAATTGG AGCTCAAAACAATAATAGCTATC TGTCAGTCTACC	
FUSC-KO-Fp	GTACTTCAACAAAAATGGAGGA ATATGAAATCCAAACAGCCCTGA TCTTTAGAACTAATG	Construction of gene cassettes for *fusC* deletion by SOE PCR
FUSC-KO-Rp	CATTAGTTCTAAAGATCAGGGCT GTTTGGATTTCATATTCCTCCATT TTTGTTGAAGTAC	
FUSC-COMP-Fp	ATGAATTAAAAGTCTACATCCAA GATTTTG	Construction of gene cassettes for *fusC* complementation by SOE PCR
FUSC-COMP-Rp	CAAAATCTTGGATGTAGACTTTT AATTCAT	
FUSC-OUT-Fp	CATTATCCTTGAAGACAGTTTATC CTGTAG	Confirmation on chromosomal integration of gene cassettes for *fusC* deletion and complementation
FUSC-OUT-Rp	CAGCAATATGATAACCACAATTA AACGTAC	
MUPA-Fp	CCTCACTAAAGGGAACAAAAGC TGGGTACCGAAAGGATGATTAAC TGATGAATAGAGCAG	Flanking primers used for amplification of gene cassettes for *mupA* deletion and complementation
MUPA-Rp	CGACTCACTATAGGGCGAATTGG AGCTCCAATATCAAATTCTCTATC TCCATATAAAC	
MUPA-KO-Fp	GAAATAAGTGATACTCTAGGAGG CTGAAAAGTTACAAACATGGCCA CTCTATTTTAGTAGAGTG	Construction of gene cassettes for *mupA* deletion by SOE PCR
MUPA-KO-Rp	CACTCTACTAAAATAGAGTGGCC ATGTTTGTAACTTTTCAGCCTCCT AGAGTATCACTTATTTC	
MUPA-COMP-	CTTAGTTGCCCTAAGTGTAATGG	Construction of gene cassettes

Fp	GAAAATGTCGCGAGTAGAAGAA GTAATCGATGTTTG	for *mupA* complementation by SOE PCR
MUPA-COMP-Rp	CAAACATCGATTACTTCTTCTACT CGCGACATTTTCCCATTACACTTA GGGCAACTAAG	
MUPA-OUT-Fp	AGTTAAAAAGTAGAACCATTAAT TTTAAATGG	Confirmation on chromosomal integration of gene cassettes for *mupA* deletion and complementation
MUPA-OUT-Rp	AATCTAATGGAAATTTTTCTAATG CTAGAG	
pIMAY-Z-Fp	GGTACCCAGCTTTTGTTCCCTTTA GTGAGG	Amplification of pIMAY-Z vector
pIMAY-Z-Rp	GAGCTCCAATTCGCCCTATAGTG AGTCG	

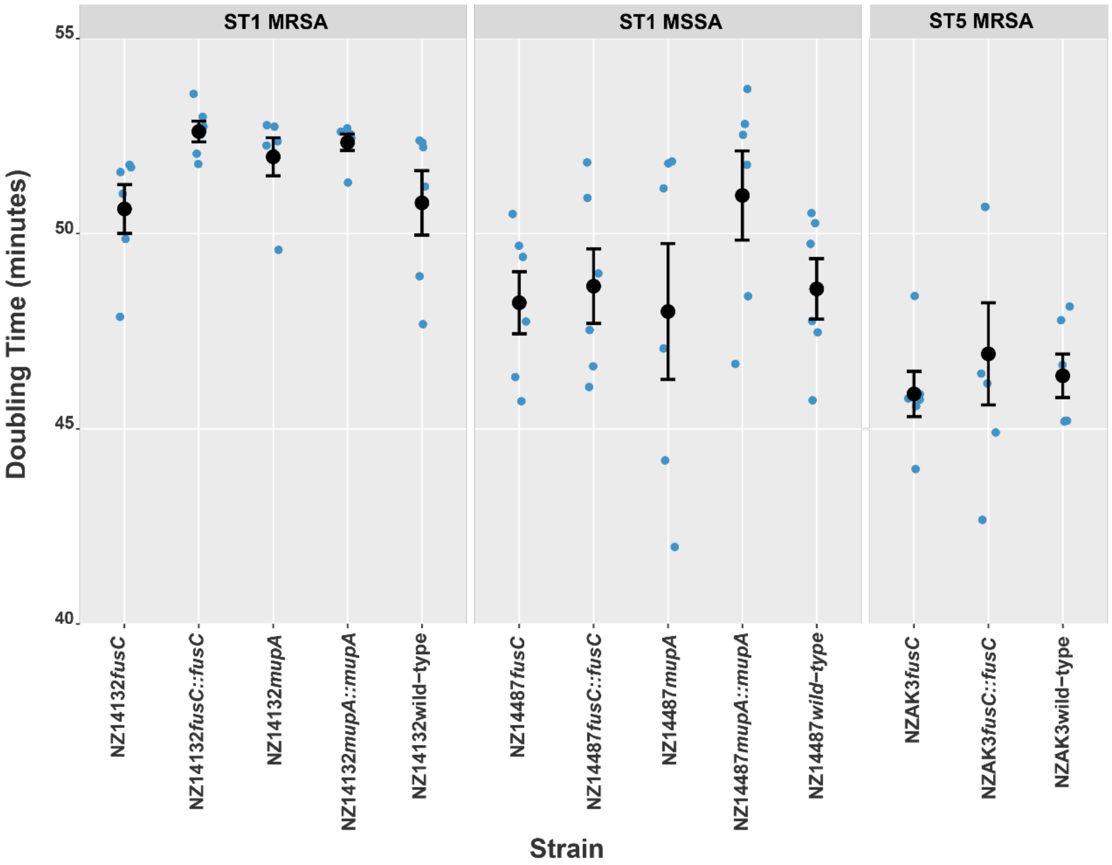

Supplementary Figure 1 – Doubling times for *S. aureus* wild-type, complemented, and mutant strains grown in BHI broth. For each strain tested, six biological replicates (blue dots) were included for determining mean doubling times (black dots) and SEM (black error bars). No significant difference ($P > 0.05$, unpaired t test) in doubling time was observed when comparing the complemented and mutant strains to their respective wild-type.
